# How Is Job Insecurity Related to Workers’ Work–Family Conflict during the Pandemic? The Mediating Role of Working Excessively and Techno-Overload

**DOI:** 10.3390/bs14040288

**Published:** 2024-03-31

**Authors:** Georgia Libera Finstad, Chiara Bernuzzi, Ilaria Setti, Elena Fiabane, Gabriele Giorgi, Valentina Sommovigo

**Affiliations:** 1 Department of Human Sciences, European University of Rome, 00163 Rome, Italy; georgialibera.finstad@unier.it (G.L.F.); gabriele.giorgi@unier.it (G.G.); 2 Department of Economics, Management, and Quantitative Methods (DEMM), University of Milan, 20122 Milan, Italy; chiara.bernuzzi@unimi.it; 3 Unit of Applied Psychology, Department of Brain and Behavioural Sciences, University of Pavia, Piazza Botta 11, 27100 Pavia, Italy; ilaria.setti@unipv.it; 4 Istituti Clinici Scientifici Maugeri, IRCCS, Department of Physical and Rehabilitation Medicine, Genova Nervi Institute, 16167 Genova, Italy; elenamaria.fiabane@icsmaugeri.it; 5 Department of Medicine and Psychology, Sapienza, University of Rome, 00185 Rome, Italy

**Keywords:** job insecurity, technostress, work–family conflict, working excessively, pandemic

## Abstract

The current labor market is characterized by drastic changes linked to the use of information and communication technologies (ICT) and post-COVID-19 transformations, which have decreased job security and job stability. As a result, the feeling of losing one’s job has become even more common among European workers. In this study, we aimed to investigate whether and how job insecurity would be related to work–family conflict during the pandemic. Online self-report questionnaires assessing job insecurity, working excessively, techno-overload, and work-to-family conflict were completed by 266 workers from Italy. Descriptive analyses, confirmatory factor analyses, and structural equation mediation models were conducted. Job insecurity was positively associated with work-to-family conflict, both directly and indirectly, as mediated by techno-overload and a tendency to work excessively. This study advances the literature, as it is the first to identify techno-overload and working excessively as parallel psychological mechanisms linking job insecurity to work–family conflict among Italian workers during the pandemic. Workers could benefit from technological workload monitoring programs, techno effectiveness, and time management training programs. Companies could also consider implementing family-friendly and digital disconnection practices.

## 1. Introduction

In recent decades, phenomena such as globalization have exerted incredible pressure on business environments, leading to changes in market structure, working patterns, and the skills deemed appealing [1]. Technology, as a substantial part of any industry, is structurally modifying the nature and organization of work, products, processes, and industrial models. The spectrum of new technologies includes, for example, the Internet of Things (IoT), big data, cloud computing, artificial intelligence (AI), automation, and robotics [2,3]. The consequences on the world of work reverberate on several levels, such as the following: the destruction and creation of employment sectors, the need for new ICT skills and generation gaps, changes in working patterns (e.g., virtual and crowd-working) and in market structures (e.g., e-commerce and sharing economy), and new psychosocial risks (e.g., technostress). For example, the available data suggest that 47% of jobs could be replaced by technology by 2030, while 27% of workers in Europe do not have sufficient digital skills. Furthermore, these trends have been irreversibly accelerated by the emergence of hybrid work models based on the use of technologies and applications for carrying out tasks remotely [4].

As for the Italian context, the available data show an alarming picture, in which 7.5% of the working population is unemployed [5]. As a result, workers experience high levels of uncertainty related to their jobs, as well as fear of pay cuts, layoff, and job loss, leading to feelings of job insecurity (JI) [6]. Even if JI can be defined according to different conceptualizations [7], it is consensually recognized as a subjective, future-focused perception of the possibility of losing one’s job [8]. Moreover, the research usually distinguishes between concerns about losing employment (i.e., quantitative JI) and concerns about losing important aspects related to the job but not the job itself (i.e., qualitative JI) [9,10]. The experience of JI is characterized by feelings of powerlessness, threat, and anxiety, representing a potential stressor [11]. JI represents a stressful factor that can lead to various cognitive, emotional, and behavioral strain reactions [7,12]. For example, research shows that JI is associated with poorer well-being; burnout; decreased performance, satisfaction, commitment, and trust; less creativity and innovation, higher levels of absenteeism, organizational counterproductive work behavior, and turnover intentions [11,12,13].

The effects of JI are not confined to work boundaries, as research has found an association with less marital satisfaction, negative effects on parenting, and greater work–family conflict (WFC) [14]. The Conservation of Resources theory (COR) offers a lens for interpreting the psychological mechanisms linking JI to outcomes in the work and family domains. According to the COR theory [15], individuals strive to obtain, retain, and protect their resources and are highly sensitive to resource losses, whereas JI endangers key resources, such as income, social support, status, and the need for skills, up to the definition of one’s identity [16,17]. In such stressful situations, meeting the demands from other domains (e.g., family) can be challenging, as is keeping negative emotions from spilling over [18]. On the other hand, as JI entails something that might happen in the future, workers may use different strategies to avoid this occurrence, as highlighted by the adaptation perspective [19].

**Figure 1:**
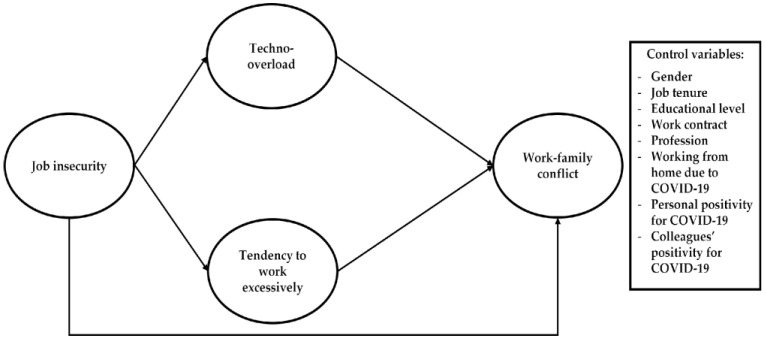
Visual representation of the expected associations.

In this study, we aim to investigate the relationship between quantitative job insecurity (JI) and work–family conflict (WFC), focusing on the behavioral consequences of potential job loss. This study focuses on quantitative, rather than qualitative, JI, particularly relevant in Italy, where it has been at the center of workers’ complaints, due to the growth of more precarious employment arrangements [20]. Drawing on the COR theory [15], the adaptation perspective [7,19,20], and the spillover theory [21] as our main frameworks, we hypothesize that JI can be associated with higher levels of WFC directly and indirectly through increased tendencies towards working excessively (the behavioral component of workaholism) and higher levels of techno-overload (see Figure 1). In doing so, this study contributes to the literature by exploring the family domain’s effects of JI, addressing the call for more research on the mechanisms underlying the associations between JI and employees’ outcomes [10]. Additionally, this study contributes to fill gaps in the research by examining contextual antecedents of workaholism, and by analyzing the techno-overload dimension, which is an understudied yet fundamental stressor in the current working context. Indeed, despite the fact that technologies are now ubiquitous in almost every sector and profession, research on technostress is still in its infancy, and most of the available data refer to traditional psychosocial risks [22].

## 2. Literature Review

### 2.1. The Relationship between Job Insecurity and Work–Family Conflict

WFC occurs when employees perceive incompatibility between work and family domains [18,21]. More specifically, it can be delineated as a type of inter-role conflict where the role demands from the work and family spheres are mutually incompatible to some degree [21]. This incompatibility can take place when the following occur: (a) the time dedicated to the demands of one role makes it difficult to fulfill the demands arising from the other role (i.e., time-based conflict); (b) the strain from the involvement in one role makes it difficult to meet the demands arising from the other role (i.e., strain-based conflict); and (c) certain behaviors demanded by one role make it difficult to satisfy the requirements arising from the other role (i.e., behavior-based conflict) [21]. The spillover theory suggests that the emotions and behaviors experienced in one domain can have an impact (either positive or negative) on the other domain. For example, negative emotions experienced at work related to JI can spill over and negatively affect the family environment, leading to conflict [18]. As for the antecedents of WFC among workers, the literature has analyzed several factors, mainly related to the characteristics of the job environment [23]. Among these, JI is particularly relevant, as the changes caused by the COVID-19 pandemic exposed workers to a generalized uncertainty about the stability of their job, increasing the share of the population at risk of losing their employment [24]. Several studies have demonstrated that employees who develop JI are more likely to experience WFC [25,26]. Following the COR perspective [15,27], the fear of a future job loss creates an uncertain situation in which employees feel that there is a threat to their valuable resources (e.g., social status and wage). People must invest further resources in order to protect against resource loss, recover from loss, and gain resources. If employees are experiencing a stressful situation, as in the case of JI, they may already be using their resources to deal with this situation, and may not be able to cope (i.e., invest further resources) with family-related demands. In a similar way, workers may try to preserve their remaining resources and protect themselves from strain by reducing the efforts dedicated to other life domains. In this scenario, workers may be less able to satisfy family requests, thereby leading to WFC [25,26]. For example, the results of the review by Mauno et al. [14] on the family-related consequences of JI highlighted a positive association between JI and WFC. Hence, we hypothesize the following:

**H1:**  *JI will be positively associated with WFC.*

### 2.2. The Mediating Role of Working Excessively in the Association between Job Insecurity and Work–Family Conflict

The behavioral aspect of workaholism, namely working excessively, is a focal point in research on the work–life interface, due to its robust positive correlation with work–family conflict, in contrast to its cognitive component, working compulsively [28]. Thus, working excessively, which refers to the individual’s drive to work extremely hard beyond what is necessary for the role [29], can serve as a behavior-based catalyst for work–family conflict, as it occurs at the detriment of fulfilling family responsibilities [30]. In this study, we hypothesize that workers may begin to engage more with their work as a job preservation strategy. As suggested by Shoss [16], employees who feel that their job position is uncertain may engage in specific behaviors with the aim of securing their employment (i.e., job preservation motivation). Similarly, the adaptation cycle model states that, when workers are faced with an undesirable condition, they engage in different kinds of behaviors aimed at changing the unpleasant situation [19]. For example, in order to demonstrate their value and relevance to the organization, workers may dedicate more time and effort to their job activities by sacrificing their free time, working more than required [9,16]. Working excessively thus becomes an instrumental impression management strategy in an attempt to reduce the perception of JI [31]. This is in line with recent empirical results. For example, the study by Spurk et al. [17] has demonstrated that career variables can explain the unique variance in workaholism, beyond the traditional personality variables. Similarly, following the COR theory, people must invest further resources in order to protect against resource loss, recover from loss, and gain resources. Moreover, resource investment gains importance in the face of potential loss [15,32]. In this case, investing in work can be seen as a task-oriented investment strategy to counteract the threat of employment loss [16].

Working excessively, in turn, can negatively influence workers’ personal sphere, leading to higher WFC. The literature has widely demonstrated that individuals with workaholic tendencies are at a higher risk of experiencing WFC [33], and this relationship is even stronger in the case of the behavioral component [20,28]. For example, individuals who tend to work excessively spend more time on work activities, paying less attention to the family environment [34]. They often miss important events, blur the lines between work and life, avoid recreational activities, and fail to meet family expectations [35]. Following the adaptation perspective, workers may start to work excessively as a behavioral response to JI, ultimately letting their work permeate their personal life. In fact, insecure workers often feel as if they “cannot afford to have a life” [19]. Similarly, following the COR theory, workers who feel insecure and invest their resources to cope with the threat of JI (i.e., working excessively) will have a drained pool of resources when it comes to taking care of family demands, leading to higher WFC [15,16,21]. For example, a recent study by Shin et al. [36] found that JI had a significant positive effect on workaholism, and this, in turn, had a significant positive effect on WFC. Therefore, we expect the following:

**H2:**  *Workers with higher levels of JI will show higher tendencies to work excessively, and this, in turn, will be related to higher levels of WFC*.

### 2.3. The Mediating Role of Techno-Overload in the Association between Job Insecurity and Work–Family Conflict

Both office and remote workers constantly rely on information and communication technologies (ICTs) to perform their work, leading to new opportunities and potential risks, such as technostress [1]. Moreover, the COVID-19 pandemic has forced every industry to rely on ICTs to continue their business [37]. ICTs are associated with stressful characteristics, such as technostress creators. Among these, techno-overload refers to the perception of increased workload and tight deadlines due to the use of technologies [22]. Indeed, technologies force employees to work longer and faster with complex technology systems and to process considerable amounts of information [38]. In our framework, we hypothesize that workers with high levels of JI will also have a greater perception of the technology-related workload, as a result of different mechanisms. Following the adaptation perspective, workers may respond to JI with strategies aimed at demonstrating their value, such as increasing their efforts at work. During the pandemic, most sectors were able to avoid the paralysis in business flow thanks to the fallback on the use of technology and new working models (depending on the level of the teleworkability of tasks, i.e., “the technical possibility of providing labor input remotely into a given economic process”). For example, in 2020, 33% of EU businesses increased the percentage of employees having remote access to ICT systems. Of these, 94% did so, at least in part, due to COVID-19 [39,40]. For workers, this drastic and sudden shift in work patterns can lead to intensified levels of technological overload [16,39,40,41]. On the other hand, it is also possible that a “regular” technological overload may be perceived as excessive by workers who are stressed by high levels of JI. In line with the COR theory, workers dealing with JI may have fewer resources to deal with technology-related demands, thus developing a higher perception of workload [15].

Techno-overload, in turn, can lead workers to develop higher levels of WFC. Previous studies have shown that stress resulting from the use of technology during work can affect employees’ private lives [42]. Moreover, techno-overload has been found to be positively linked to WFC [43,44]. Drawing on the COR theory [15], this represents a stressful situation in which employees use their valuable resources to deal with job-related pressures, thus reducing the remaining resources for the family domain. Accordingly, techno-overload has been found to be strongly associated with workload [45], which represents one of the most studied antecedents of WFC [46]. Recent empirical evidence also supports the positive association between techno-overload and WFC [43,44]. Hence, we hypothesize the following:

**H3:**  *Workers with higher levels of JI will show higher levels of techno-overload, and this, in turn, will be related to higher levels of WFC.*

## 3. Materials and Methods

### 3.1. Participants and Procedure

Following the acquisition of research ethical approval from the Research Ethics Committee of the University of Pavia (Protocol no. 068/20), we collected data using a computerized survey developed using a spreadsheet in Google Sheets. The data collection period spanned from December 2020 to February 2021. We disseminated the survey link through various social network platforms, including LinkedIn, Instagram, Twitter, Facebook, and WhatsApp. The link was accompanied by a cover letter explaining our research goals, who could take part in our research (i.e., employees over the age of 18 who had provided informed consent), and why the respondents should voluntarily take around 20 min of their time to complete the survey. The cover letter also informed the respondents about their anonymity and the confidentiality of the responses. A total of 297 participants completed the online survey. We excluded 30 cases because of incomplete answers (i.e., less than 60% of the questionnaire), and 6 cases because they were multivariate outliers, leaving a sample size of 261 usable cases. Most of the participants were women (62.80%), with an average job tenure of up to 5 years (44.00%). Most of the respondents had a master’s degree (48.70%) and an employment contract (74.70%). In addition, most of the participants were practicing intellectual professions (42.50%) and had begun to work from home due to the pandemic (56.30%). Among them, 9.20% were positive for COVID-19, and 54.20% had colleagues who had been diagnosed with the virus (see Table 1).

### 3.2. Measurements

Job insecurity was measured with a single item, which was previously used by Gasparro et al. [48] to investigate the fear of job loss in the COVID-19 pandemic context. The respondents reported how secure they felt about their job in their current work environment due to the pandemic (i.e., How secure do you feel about your job or career prospects in your current workplace due to the COVID-19 outbreak?) using a five-point Likert scale, ranging from 1 = very secure to 5 = not at all secure.

Techno-overload was measured with four items from the Italian Technostress Creators Scale [41]. The participants reported how frequently they felt forced by technology to do more work than they could handle (e.g., I am forced to change my work habits to adapt to new technologies, α = 0.86) using a five-point Likert-type scale (1 = strongly disagree, 5 = strongly agree).

Working excessively was measured using the five-item subscale from the Italian version of the Dutch Work Addiction Scale [49]. The participants reported how frequently they tended to work excessively hard (e.g., I stay busy and keep many irons in the fire, α = 0.72) on a five-point Likert-type scale (1 = never or almost never, 5 = almost always or always).

Work–family conflict was studied with the five-item subdimension from the Work–Family Conflict scale [50]. The respondents reported their agreement level with statements describing situations wherein the role pressures from the work made it difficult to meet requests from the family domain (e.g., My job produces strain that makes it difficult to fulfill family duties, α = 0.89) on a seven-point Likert-type scale (1= completely disagree, 7= completely agree).

In terms of control variables, we controlled for gender (0 = man, 1 = woman), job tenure (1 = up to 5 years; 2 = from 5 to 15 years; 3 = more than 15 years), educational level (1 = elementary school; 2 = high school graduate; 3 = bachelor’s degree; 4 = master’s degree; 5 = PhD or higher professional training), work contract (0 = employment, 1 = self-employment), type of profession (i.e., dummy variables for intellectual and technical professions), and working from home during the health emergency (i.e., Currently, are you working remotely due to COVID-19? 0 = no, 1 = yes). We also accounted for personal factors such as whether the respondents had been diagnosed with COVID-19 themselves, as well as the positivity of their colleagues for the virus. We included gender, as previous evidence suggests JI may differ in female and male employees, with two opposing perspectives [11]. On the one hand, men could experience less distress, because they usually have higher career mobility [11,51]. On the other hand, women could show lower levels of JI, because they place more value on other responsibilities (e.g., family roles). As for WFC, women typically experience higher levels of conflict [52]. According to gender role theory (or sensitization perspective), women are more sensitive to interference in the family domain, leading to higher levels of WFC. This is also in line with the “rational view,” according to which conflicts are experienced in the domain in which the most time is spent. Evidence also suggests that women experience a greater tendency to work excessively, as well as certain types of workaholic behaviors (e.g., perfectionism) [53]. Similarly, men and women appear to differ in their technostress experience, with men reporting higher levels of techno-overload and techno-invasion and women reporting higher levels of techno-complexity and techno-uncertainty [54]. As for the relationship between age and WFC, the evidence suggests a possible relationship with lower levels of WFC for younger and older employees, as, at different life stages, individuals can experience high pressure in both domains (e.g., young adults manage challenging career expectations while caring for young children) [55]. Even when analyzing JI, the research suggests a possible association between higher levels of JI and older workers, as well as workers with a longer tenure. A longer tenure is associated with stronger commitment and identification feelings, leading to worse reactions to insecurity [11,51,56]. The available research also highlights that workaholism could be influenced by age, suggesting a declining pattern. This could be due to the acquisition of better coping strategies or quitting the job [57,58]. Older workers also report greater techno-overload and tend to be less familiar with technologies, reporting greater difficulties managing a constant flow of information [59] and ignoring interruptions [60]. We included the level of education, as this variable shapes future occupational opportunities, and this, in turn, influences the type of demands (e.g., working hours) and resources (e.g., earnings and benefits) that contribute to WFC [61]. Regarding JI, the evidence highlights varying results, with some studies showing that workers with higher education feel safer in their jobs and perceive more opportunities, while other studies show the opposite trend [62,63,64]. Similarly, high-level professionals tend to report higher levels of working excessively and compulsively, as do employees working in certain sectors (e.g., communication, consultancy, agriculture, construction, and trade) [65]. People with higher educational levels are also naturally more exposed to ICTs, thus being able to adapt more easily [66]. In this case, the research tends to show a negative association between formal higher education and levels of technostress [38,67,68]. Regarding the type of contract, the research shows conflicting results, highlighting how self-employed workers may have resources such as autonomy and flexibility to better balance their work and family domains, while, on the other hand, longer working hours and precariousness could lead to tension and WFC [69,70]. Studies also show that self-employed workers tend to report higher tendencies to work excessively compared to employees [65]. Regarding JI, scholars suggest that self-employed workers could perceive precariousness in terms of employment insecurity [71]. Finally, we included working from home, as this can blur the boundaries between the work and personal spheres, leading to possible overlaps and longer working hours, which can translate into higher levels of WFC and workaholism [72,73]. Some research also suggests a positive association between working from home and JI. Indeed, employees can develop feelings of alienation and career concern related to the lack of visibility and direct connection with the work environment [73]. Remote workers also report higher levels of overload, perceiving greater pressure and the expectation of flexibility [38,74], especially in the pandemic period [41].

### 3.3. Statistical Analyses

After performing a power analysis using G*Power [75], we examined our data for multicollinearity and normality, detecting outliers using the *p* < 0.001 criterion for Mahalanobis distance. Following previous methodologists’ recommendations [76], as reliability measures, Cronbach’s alpha, McDonald’s omega, composite reliability (CR), and average variance extracted (AVE) were calculated in SPSS 25 [77] and JASP [78]. Next, we explored the data for descriptive statistics and correlations using SPSS 25. Then, we performed confirmatory factor analyses (CFAs) with the maximum likelihood method, comparing the measurement model with a series of alternative models using Mplus version 8 [79]. We then evaluated the potential impact of common method bias using the following: (a) Harman’s single-factor test with unrotated factor solutions [80]; and (b) the unmeasured latent method factor technique, by allowing indicators of our expected CFA to load on their corresponding constructs and the method factor [81]. Then, we conducted our expected parallel mediation model using bootstrapping tests and a bias-corrected 95% confidence interval (CI) with a resampling procedure of 1000 bootstrap samples from the full sample. The indirect effects were considered significant when the ***p***-value was less than or equal to 0.05 and the 95% confidence interval from the bootstrap analysis did not include 0 [82]. The model fits were evaluated by examining the magnitude and statistical significance of the factor loadings, the root mean square error of approximation (RMSEA), the standardized root mean square residual (SRMR), the comparative fit index (CFI), and the Tucker–Lewis index (TLI) [83,84].

## 4. Results

### 4.1. Measurement Reliability and Validity

The results indicated that the factor loadings of all of the items on their corresponding constructs were above 0.5, showing at least a medium correlation with the relevant construct (techno-overload: 0.82–0.86; working excessively: 0.63–0.74; and work–family conflict: 0.77–0.87). Moreover, the results showed that the composite reliability coefficients for the study’s constructs ranged from 0.83 to 0.92. In support of convergent validity, the AVE values for the constructs ranged from 0.51 to 0.70. 

### 4.2. Descriptive Analyses

The results of the power analysis for a multiple regression analysis, including 11 predictors, using an alpha of 0.05, a power of 0.95, and a medium effect size, indicated that a minimum sample size of 178 subjects was necessary, suggesting the adequacy of our sample size. The data underwent an examination for outliers, multicollinearity, and normality. Seven multivariate outliers were removed using the *p* < 0.001 criterion for Mahalanobis distance. Additionally, all of the correlations among the study constructs aligned with the expected direction (see Table 2). 

### 4.3. Confirmatory Factor Analyses and Assessment of Common Method Bias

The results from the CFA (see Table 3) have indicated that the fit indices of the three-factor model were satisfactory (χ2 [74] = 147.62, RMSEA = 0.05, SRMR = 0.05, CFI = 0.95, TLI = 0.94). This model has demonstrated superior fit indices compared to all of the alternative models, thereby confirming the distinctiveness of the study constructs. Additionally, the Harman’s single-factor test results revealed that the first factor accounted for only 35.53% of the variance. Moreover, the unmeasured latent method factor explained 24.80% of the total variance, suggesting that the common method variance was unlikely to be a significant concern in our study.

### 4.4. Hypotheses Testing

**Figure 2:**
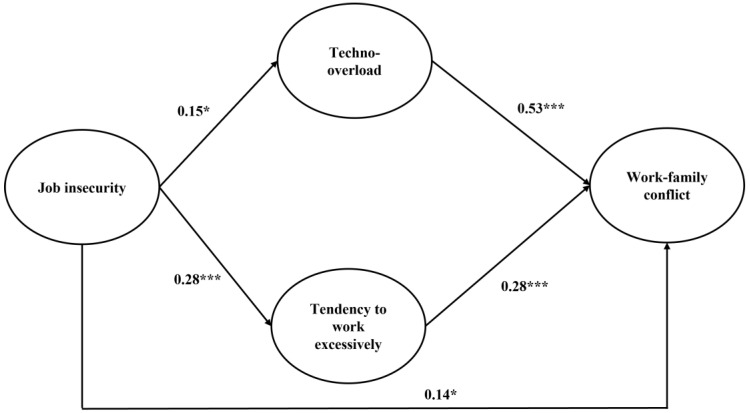
Path coefficients for the parallel mediation model. Note. * = *p* < 0.05; *** = *p* < 0.001.

In the hypothesized parallel mediation model (χ2 [185] = 335.64, RMSEA = 0.06, SRMR = 0.06, CFI = 0.91, TLI = 0.90; see Table 4 and Figure 1), job insecurity was positively related to work–family conflict (β = 0.14, SE = 0.07, *p* < 0.05), working excessively (β = 0.15, SE = 0.07, *p* < 0.05), and tecno-overload (β = 0.28, SE = 0.06, *p* < 0.001). Additionally, working excessively (β = 0.53, SE = 0.07, *p* < 0.001) and techno-overload (β = 0.28, SE = 0.08, *p* < 0.001) were positively related to work–family conflict and mediated, in parallel, the relationship between job insecurity and work–family conflict (working excessively: β = 0.08, SE = 0.04, *p* < 0.05; tecno-overload: β = 0.08, SE = 0.03, *p* < 0.001) (see Figure 2). Among the control variables, work contract was statistically significantly and positively related to working excessively (β = 0.18, SE = 0.08, *p* < 0.05), whilst job tenure (β = 0.17, SE = 0.06, *p* < 0.01) and working from home (β = 0.25, SE = 0.07, *p* < 0.01) were statistically significantly and positively associated with techno-overload. Additionally, being a woman was positively related to job insecurity (β = 0.15, SE = 0.07, *p* < 0.05). Overall, technostress was related to work–family conflict, both directly and indirectly, as mediated, in parallel, by working excessively and techno-overload. It is important to note that the indirect effects of working excessively (β = 0.08, SE = 0.04, *p* < 0.05) and techno-overload (β = 0.08, SE = 0.03, *p* < 0.01) remained statistically significant also in the parallel mediation model (χ2 [86] = 175.86, RMSEA = 0.06, SRMR = 0.07, CFI = 0.94, TLI = 0.93) without the inclusion of the control variables. Hence, Hypotheses 1, 2, and 3 were confirmed.

## 5. Discussion

In this study, we investigated what happens beyond the work context and how workers respond to JI, that is, how JI is associated with other crucial domains and what strategies workers employ to deal with this threat. Specifically, we focused on the behavioral consequences of JI as possible underlying mechanisms. The results confirmed our hypotheses, showing that JI is associated with WFC, both directly and indirectly, through working excessively and techno-overload. 

Drawing on the COR theory [15], and based on previous empirical evidence [14], JI represents a job stressor, as it threatens workers’ valuable resources by triggering worries about their employment status and negative emotions (e.g., fears of losing one’s job), thereby leaving them with lower resources to meet the demands from the family domain. Accordingly, the literature has demonstrated that JI is predictive of WFC [25,26]. When experiencing JI, workers may react by investing their energy into their job to perform better in order to show their value [15]. Thus, if workers perceive that their employment is at risk, they will adopt a series of actions aimed at avoiding the threat (i.e., increasing their work efforts), thus initiating a potential dysfunctional pattern [16,19]. From a career perspective, workaholism can then be seen as a coping strategy in response to economic stressors [17]. In an attempt to secure their job position, workers may encounter a resource drain process that leaves them with fewer resources to satisfy family-related demands. This is consistent with previous studies showing a positive association between behavioral reactions, such as working excessively, and WFC [31]. Drawing on the COR theory [27,32], workers dealing with JI may also have fewer resources to deal with technology-related demands, thus developing a higher perception of needing to work at a quicker pace and for longer durations because of ICTs (i.e., techno-overload). This depletion may leave them without enough resources to take care of their family demands, which may result in greater WFC [36]. Accordingly, the previous research found that workers invested considerable time and effort into managing technologies during the pandemic, resulting in higher levels of technology-related workload [41].

Moreover, working from home due to COVID-19 was positively related to techno-overload. A substantial part of the Italian working population began working remotely for the first time during the pandemic, without receiving any specific training [30,41]. This guaranteed a continuous flow of information and may have created the unrealistic expectation of having to process this amount of data in real-time [85], thereby increasing the risk for remote workers to experience greater techno-overload, as demonstrated in previous studies [30,41]. Furthermore, the positive association between job tenure and techno-overload is understandable, based on the aging workforce literature, as job tenure is an indicator of age. According to this field of research, older workers are at a higher risk of experiencing techno-overload because of age-related reductions in the physical capabilities essential for technology utilization and lower digital literacy than their younger counterparts [30]. Moreover, being a woman was positively related to JI. This is understandable, as women often occupy less protected jobs and were employed in sectors affected by the crisis. For instance, the number of women who lost their jobs in 2020 was more than double that of their male counterparts [24]. Finally, consistent with prior research [65], being self-employed was positively related to working excessively. Thus, individuals in entrepreneurial jobs are characterized by achievement-related traits that are predictive of workaholism. Additionally, quantitative work overload and working excessively long hours are highly prevalent among self-employed individuals, making them a risk group for workaholism [65].

This study moves the research a step forward in terms of management literature, as it clarifies the psychological mechanisms linking JI to WFC, thereby providing new insights into managing human resources effectively during uncertain economic times.

### 5.1. Limitations and Future Research Directions

Despite the precautions taken, this study has some limitations that should be addressed. The cross-sectional design of our study does not allow for causal inferences. Future studies should employ a longitudinal design in order to further analyze the direction of the relationships. In addition, we relied on self-report measures, increasing the risk of common method bias. Future studies could use multi-method data, such as intersubjective and objective evaluations. Furthermore, we employed a non-probabilistic sampling method, thereby constraining the generalizability of the findings. Subsequent research endeavors should aim to validate these results using a more diverse and representative sample. Moreover, we focused on quantitative JI, while future research could compare the effects of quantitative JI with those of qualitative JI, thus providing more nuanced information. Finally, our analysis did not consider any moderator. Our framework could be integrated with the analysis of different individual and contextual variables that can influence the relationships between the constructs. 

### 5.2. Practical Implications and Conclusion

Given the detrimental effects of JI on WFC, the government and companies should take steps to reduce JI in the first place. Since previous research has found that flexicurity policies can reduce stress reactions to JI [86], policies supporting the capability of employed and self-employed workers’ to have occupational mobility, and, simultaneously, the creation of a robust social safety net, could be introduced. Concurrently, companies could implement participatory organization-level intervention programs wherein employees and managers can participate in prioritization and planned activity workshops to address the JI resulting from intra- and/or extra-organizational changes [87]. Interventions could also be designed and implemented to strengthen the perceptions of organizational resources (e.g., good communication) and non-contextual resources (e.g., career management skills) [11]. In parallel, technological workload monitoring mechanisms should be introduced together with techno-effectiveness training to increase the mastery of ICTs, and time management training programs, as well as reverse mentoring in the use of ICTs, should also be introduced, especially for older remote workers [88]. For example, stress management programs could be useful to increase awareness about the reasons for and risks of working excessively, reinforcing the individual’s ability to psychologically detach in order to have moments of “recovery” and digital disconnection. However, this should be supported by the implementation of family-friendly practices and the creation of an organizational culture that values disconnection and discourages the expectation of constant availability [22,41]. To conclude, we hope that this study will encourage future research on the spillover effects of job insecurity in order to provide new insights into how to design interventions promoting a healthy work–life interface, even during uncertain times. 

## Figures and Tables

**Table 1:** Descriptive statistics regarding the sample (*n* = 261).

Variable	*n*	%
Gender (%)		
Women–Men	164–97	62.8–37.2
Job tenure (%)		
<5 years	115	44.0
5–15 years	79	30.3
>15 years	67	25.7
Education (%)		
High school certificate or lower	92	35.2
Bachelor’s degree	42	16.1
Master’s degree or higher	127	48.7
Occupation * (%)		
Intellectual profession	111	42.5
Technical profession	43	16.5
Service profession	107	41.0
Work contract (%)		
Employment–Self-employment	195–66	74.7–25.3
Working from home due to COVID-19 (%)		
Yes–No	147–114	56.3–43.7
Personal positivity for the COVID-19 virus (%)		
Yes–No	237–24	90.8–9.2
Colleagues’ positivity for the COVID-19 virus (%)		
Yes–No	142–119	54.2–45.8

* The classification of occupations is based on the Labor Force Survey (LFS) employment series [47].

**Table 2:** Intercorrelations and descriptive statistics among the study variables (*n* = 261).

	M	SD	CR	AVE	ω	Ske.	Kur.	1	2	3	4	5	6	7	8	9	10	11	12	13
1. Job insecurity	2.87	1.30	-	-	-	0.14	−1.03	-												
2. Techno-overload	2.33	1.13	0.90	0.70	0.86	0.66	−0.53	0.23 **^,a^	**0.86**											
3. Working excessively	2.85	0.69	0.83	0.51	0.73	−0.28	−0.53	0.13 *^,a^	0.23 **^,a^	**0.72**										
4. Work–family conflict	3.50	1.61	0.92	0.69	0.89	0.24	−0.82	0.28 **^,a^	0.42 **^,a^	0.45 **^,a^	**0.89**									
5. Gender	-	-	-	-	-	-	-	0.16 **^,b^	0.02 ^b^	0.12 ^b^	0.10 ^b^	-								
6. Job tenure	-	-	-	-	-	-	-	−0.02 ^c^	0.14 **^,c^	−0.05 ^c^	0.01 ^c^	0.05 ^b^	-							
7. Education	-	-	-	-	-	-	-	0.05 ^c^	0.14 **^,c^	0.10 *^,c^	0.10 *^,c^	0.35 *^,b^	−0.09 ^b^	-						
8. Work contract	-	-	-	-	-	-	-	−0.09 ^b^	0.01 ^b^	0.12 *^,b^	−0.04 ^b^	−0.21 **^,d^	0.03 ^b^	−0.06 ^b^	-					
9. Intellectual professions	-	-	-	-	-	-	-	0.02 ^b^	0.14 *^,b^	−0.02 ^b^	0.05 ^b^	0.18 **^,d^	−0.02 ^b^	0.50 **^,b^	−0.04 ^d^	-				
10. Technical professions	-	-	-	-	-	-	-	0.03 ^b^	0.05 ^b^	−0.01 ^b^	0.12 *^,b^	0.01 ^d^	−0.09 ^b^	−0.05 ^b^	−0.02 ^d^	−0.38 **^,d^	-			
11. Service professions	-	-	-	-	-	-	-	0.04 ^b^	−0.18 **,^b^	0.02 ^b^	−0.15 *^,b^	0.18 **^,d^	−0.08 ^b^	−0.46**,^b^	−0.05 ^d^	−0.72 **,^d^	−0.37 **^,d^	-		
12. Personal positivity	-	-	-	-	-	-	-	−0.02 ^b^	0.05 ^b^	0.01 ^b^	0.12 ^b^	0.02 ^d^	−0.09 ^b^	−0.03 ^b^	−0.01 ^d^	0.02 ^d^	0.14 *^,d^	−0.13 *^,d^	-	
13. Colleague’s positivity	-	-	-	-	-	-	-	0.01 ^b^	0.10 ^b^	−0.05 ^b^	0.07 ^b^	0.09 ^d^	−0.01 ^b^	0.11 ^b^	−0.20 **^,d^	0.13 *^,d^	0.04 ^d^	−0.16 *^,d^	0.13 *^,d^	-
14. Working from home	-	-	-	-	-	-	-	−0.01 ^b^	0.23 **^,b^	0.03 ^b^	0.10 ^b^	0.13 *^,d^	0.05 ^b^	0.46 **^,b^	−0.07 ^d^	0.43 **^,d^	−0.18 **^,d^	−0.29 **^,d^	−0.09 ^d^	0.14 *^,d^

Note. Boldfaced numbers on the diagonal represent Croncach’s alphas; M = mean; SD = standard deviation; CR = composite reliability; AVE = average variance extracted; ω = McDonald’s omega; Ske. = skewness; Kur. = kurtosis; * = *p* < 0.05; ** = *p*< 0.01; ^a^ = Pearson’s correlation coefficient; ^b^ = Spearman’s rho correlation coefficient; ^c^ = Kendall’s coefficient of rank correlation tau-sub-b; ^d^ = Phi coefficient obtained from contingency table. Gender: 0 = man, 1 = woman; Job tenure: 1 = up to 5 years of experience, 2 = from 6 to 15 years of experience, 3 = more than 15 years of experience; Work contract: 0 = employment, 1 = self-employment; Intellectual professions: 0 = other professions, 1 = intellectual professions; Technical professions: 0 = other professions, 1 = technical professions; Service professions: 0 = other professions, 1 = service professions; Personal positivity for the COVID-19 virus: 0 = no, 1 = yes; Colleague’s positivity for the COVID-19 virus: 0 = no, 1 = yes; Working from home: working from home due to COVID-19 pandemic, 0 = no, 1 = yes.

**Table 3:** Fit indices for the six-factor model and the alternative models (*n* = 261).

Model	χ2	df	*p*	RMSEA	90%RMSEA	SRMR	CFI	TLI
Three-factor model_meth ^f^	97.75	60	0.00	0.05	[0.03, 0.06]	0.04	0.98	0.96
Three-factor model ^e^	147.62	74	0.00	0.05	[0.05, 0.08]	0.05	0.95	0.94
Two-factor model3 ^d^	259.69	76	0.00	0.10	[0.08, 0.11]	0.07	0.88	0.86
Two-factor model 2 ^c^	477.41	76	0.00	0.14	[0.13, 0.15]	0.10	0.74	0.69
Two-factor model 1 ^b^	376.25	76	0.00	0.12	[0.11, 0.14]	0.12	0.80	0.77
One-factor model ^a^	591.28	77	0.00	0.16	[0.15, 0.17]	0.12	0.67	0.61

χ2 = chi square; df = degree of freedom; RMSEA = root mean square error of approximation; SRMR = standardized root mean square residuals; CFI = comparative fit index; TLI = Tucker–Lewis index. ^a^ All indicators load on a single factor. ^b^ Work excessively and techno-overload on one factor; work–family conflict loads on a second factor. ^c^ Work–family conflict and techno-overload on one factor; work excessively load on a second factor. ^d^ Work–family conflict and work excessively on one factor; techno-overload on a second factor. ^e^ Work-family conflict, work excessively, and techno-overload load on their respective factors. ^f^ Previous model with the inclusion of a common method latent variable, on which make all the items loaded.

**Table 4:** Fit indices and standardized direct and indirect effects for the parallel mediation model.

Model (Outcome)	χ2	df	*p*	RMSEA	SRMR	CFI	TLI
Model 1	335.64	185	0.000	0.06 [0.05,0.07]	0.06	0.91	0.90
**Standardized direct and indirect effects**							
Effects-Model 1		*Est.*		*S.E.*		*95% CI*	
Gender → Job insecurity		0.15 *		0.07		[0.04, 0.26]	
Job tenure → Job insecurity		−0.04		0.06		[−0.13, 0.06]	
Educational level → Job insecurity		−0.01		0.09		[−0.14, 0.14]	
Work contract → Job insecurity		−0.05		0.06		[−0.16, 0.06]	
Intellectual profession → Job insecurity		0.03		0.08		[−0.01, 0.16]	
Technical profession → Job insecurity		0.02		0.07		[−0.10, 0.14]	
Personal positivity for the COVID-19 virus → Job insecurity		−0.02		0.08		[−0.15, 0.11]	
Colleagues positive for the COVID-19 virus → Job insecurity		0.01		0.06		[−0.09, 0.12]	
Working from home due to COVID-19 → Job insecurity		−0.04		0.08		[−0.17, 0.08]	
Gender → Techno-overload		−0.08		0.07		[−0.19, 0.03]	
Job tenure → Techno-overload		0.17 **		0.06		[0.06, 0.28]	
Educational level → Techno-overload		0.13		0.09		[−0.02, 0.28]	
Work contract → Techno-overload		0.01		0.07		[−0.10, 0.13]	
Intellectual profession → Techno-overload		−0.01		0.09		[−0.15, 0.14]	
Technical profession → Techno-overload		0.12		0.07		[−0.01, 0.24]	
Personal positivity for COVID-19 → Techno-overload		0.06		0.06		[−0.05, 0.16]	
Colleagues positive for COVID-19 → Techno-overload		0.03		0.07		[−0.08, 0.14]	
Working from home due to COVID-19 → Techno-overload		0.25 **		0.07		[0.12, 0.37]	
Gender → Working excessively		0.14		0.10		[−0.02, 0.30]	
Job tenure → Working excessively		−0.09		0.08		[−0.22, 0.03]	
Educational level → Working excessively		0.12		0.09		[−0.03, 0.27]	
Work contract → Working excessively		0.18 *		0.08		[0.05, 0.31]	
Intellectual profession → Working excessively		−0.14		0.09		[−0.28, 0.01]	
Technical profession → Working excessively		−0.02		0.08		[−0.15, 0.11]	
Personal positivity for COVID-19 → Working excessively		0.04		0.09		[−0.11, 0.18]	
Colleagues positive for COVID-19 → Working excessively		−0.05		0.08		[−0.17, 0.08]	
Working from home due to COVID-19 → Working excessively		0.06		0.09		[−0.09, 0.20]	
Gender → Work–family conflict		−0.02		0.06		[−0.12, 0.07]	
Job tenure → Work–family conflict		0.02		0.06		[−0.08, 0.11]	
Educational level → Work–family conflict		0.03		0.07		[−0.08, 0.15]	
Work contract → Work–family conflict		−0.10		0.06		[−0.20, −0.01]	
Intellectual profession → Work–family conflict		0.03		0.08		[−0.09, 0.16]	
Technical profession → Work–family conflict		0.13		0.07		[−0.02, 0.13]	
Personal positivity for COVID-19 → Work–family conflict		0.08		0.06		[−0.02, 0.13]	
Colleagues positive for COVID-19 → Work–family conflict		0.03		0.06		[−0.06, 0.17]	
Working from home due to COVID-19 → Working excessively		0.08		0.05		[−0.11, 0.11]	
Job insecurity → Work–family conflict		0.14 *		0.07		[0.03, 0.26]	
Job insecurity → Working excessively		0.15 *		0.07		[0.03, 0.27]	
Job insecurity → Techno-overload		0.28 ***		0.06		[0.17, 0.38]	
Working excessively → Work–family conflict		0.53 ***		0.07		[0.41, 0.65]	
Techno-overload → Work–family conflict		0.28 ***		0.08		[0.15, 0.41]	
Job insecurity → Techno-overload → Work–family conflict		0.08 *		0.04		[0.03, 0.12]	
Job insecurity → Working excessively → Work–family conflict		0.08 **		0.03		[0.01, 0.14]	
Total effects on Work–family conflict		0.30 ***		0.07		[0.19, 0.42]	

Note. * = *p* < 0.05; ** = *p* < 0.01; *** = *p* < 0.001; df = degree of freedom; RMSEA = root mean square error of approximation df = degree of freedom; RMSEA = root mean square error of approximation; SRMR = standardized root mean square residuals; CFI = comparative fit index; TLI = Tucker–Lewis index.

## Data Availability

The data used for this study are available from the corresponding author upon reasonable request.
